# Compaction Characteristics and Permeability of Expansive Shale Stabilized with Locally Produced Waste Materials

**DOI:** 10.3390/ma15062138

**Published:** 2022-03-14

**Authors:** Muhammad Rehan Hakro, Aneel Kumar, Zaheer Almani, Mujahid Ali, Roman Fediuk, Sergey Klyuev, Alexander Klyuev, Linar Sabitov, Dina Fathi

**Affiliations:** 1 Department of Civil Engineering, Mehran University of Engineering and Technology, Jamshoro 76062, Sindh, Pakistan; aneel.kumar@faculty.muet.edu.pk (A.K.); zaheer.almani@faculty.muet.edu.pk (Z.A.); 2 Department of Civil and Environmental Engineering, Universiti Teknologi PETRONAS, Bandar Seri Iskandar 31750, Perak, Malaysia; 3 Polytechnic Institute, Far Eastern Federal University, 690922 Vladivostok, Russia; 4 Peter the Great St. Petersburg Polytechnic University, 195251 St. Petersburg, Russia; 5 Department of Theoretical Mechanics and Strength of Materials, Belgorod State Technological University Named after V.G. Shukhov, 308012 Belgorod, Russia; klyuyevav@yandex.ru; 6 Kazan Federal University, 420008 Kazan, Russia; sabitov-kgasu@mail.ru; 7 Kazan State Power Engineering University, 420066 Kazan, Russia; 8 Structural Engineering and Construction Management Department, Faculty of Engineering and Technology, Future University in Egypt, New Cairo 11845, Egypt; dinamohamed@fue.edu.eg

**Keywords:** soil compaction, soil stabilization, environmental pollution, waste materials, expansive clay, permeability

## Abstract

Waste is available in an abundant form and goes to landfill without any use, creating threats to the environment. Recent and past studies have used different types of waste to stabilize soil and reduce environmental impacts. However, there is a lack of studies on the combined use of marble dust, rice-husk ash, and saw dust in expansive shale soil. The current study tries to overcome such a gap in the literature, studying the effect of marble dust, rice-husk ash, and saw dust on expansive shale’s compaction characteristics and permeability properties. According to unified soil classification and the AAHTO classification system, the geotechnical properties of natural soil are classified as clay of high plasticity (CH) and A-7-5. Several tests are performed in the laboratory to investigate the compaction characteristics and permeability properties of expansive shale. Moreover, permeability apparatus is used to investigate the permeability properties of soil. In addition, due to the accuracy of the apparatus, the conventional apparatus has been partly modified. The experimental results show that the addition of waste to the soil has significantly improved soil stabilization, increasing permeability and decreasing plasticity indexes. In addition, there is a gradual decrease in the dry density of soil and an increase in the permeability of stabilized soil. Based on the outcomes of the current study, it claims and concludes that these waste materials can be used as soil stabilizers or modifiers, instead of being dumped in landfill, which will provide a green, friendly, and sustainable environment. The current study recommends that future researchers use various wastes in the concrete and soil to improve their compaction and mechanical properties.

## 1. Introduction

Soil is a complex, varied, and unpredictable material that is left to nature’s whims. Because of environmental, loading, and drainage differences, soil quality varies between sites and within areas. Clay shale comprises 50 to 70 percent sedimentation rock [1,2]. It exhibits brittleness and low durability [3,4]. Clay shale is prone to weathering, which results in fast degradation of its geotechnical characteristics, strength, and durability [5,6,7,8]. Some of the difficulties resulting from expansive shale’s behavior are: destabilizing fill material, a reduction in the bearing capacity of shallow, deep foundations, slope stability, plumbing, and subsidence [9,10,11,12,13,14].

Soil’s characteristics are determined by both its type and the environment in which it grows. Due to the vast volume of soil involved, and the fact that it is not open for inspection at greater depth for the foundations of various projects, transporting soils is not economically feasible in comparison to other construction materials, such as concrete or steel. Civil engineers are occasionally tasked with constructing a facility on a site selected for reasons other than soil conditions. As a result, it is becoming increasingly important for the engineer to understand the extent to which the soil’s technical features can be improved or alternative structures can be used for building at the given location [15,16,17].

Expansive clay soils spread over large areas all over the world. The properties of these problematic soils at shallow depth show volume change from variations in soil moisture, due to seepage of water into the soils during rains [18,19]. These soils contain the clay mineral montmorillonite, which causes swelling and shrinking in the ground due to increasing and decreasing water content [20,21]. Damages to civil structures (e.g., railways, road networks, buried pipelines, and other lifeline facilities) due to soil swelling and shrinking soil problems can be considered to be the costliest natural hazard in some countries. In addition to the challenges associated with expansive soils, another issue, because of industrialization, is that waste generated by companies poses serious environmental problems and requires a large amount of land for disposal in all emerging and developed countries.

Clay soils are often stiff when dry but lose stiffness when saturated with water. Soft clays have a limited bearing capacity and are highly compressible. Waste stone sludge from slab stone processing and stone washing plants was recycled in Roohbakhshan and Kalantari’s [22] study of lime stabilization in clayey soil. In the laboratory, the efficiency of waste stone powder and lime in stabilizing fine-grained clayey soil was tested.

Gypsum (CaSO_4_·2H_2_O) is employed as one of the soil stabilizing agents in a study by Reethu et al. [23] to stabilize clayey soil and attain better strength in a short period of time. Experiments were designed to evaluate the properties of clayey soil after adding different percentages of gypsum to the existing soil; namely, 2 percent, 4 percent, 6 percent, and 8 percent. The Atterberg limits, specific gravity test, and the standard Proctor test were all performed on clayey soil mixed with gypsum.

The research of Alzaidy [24] assessed an experimental investigation for stabilizing a clayey soil with eggshell powder as a replacement for commercial lime and plastic waste strips, in order to lessen the brittleness of the soil stabilized by eggshell powder, as well as the effect on the soil’s engineering properties. To determine the best percentage of each addition, nine groups of stabilized soil samples were made using three different proportions of eggshell powder (2 percent, 5 percent, and 8 percent by weight of dry soil) and plastic waste strips (0.25 percent, 0.5 percent, and 1 percent by weight of dry soil). Compaction, unconfined compression, swelling potential, direct shear, and California bearing ratio tests were used in the investigation.

When expansive soils are used as a subgrade layer, mitigation of the swelling and shrinking potential of expansive soils is a significant design feature of pavement structures. Seasonal moisture variations cause extensive heaving and shrinking of expansive subgrade soils, resulting in the breakdown of the upper pavement structure. In an experimental study of Dayioglu et al. [25], expansive clay was combined with fly ash and lime. On samples cured for various times (0, 7, and 28 days), swelling pressure (SWP) and unconfined compressive strength (UCS) tests were performed.

The waste production from any industry is a part of the industry’s functioning. The fundamental component of any manufacturing or production enterprise is the waste management [26,27,28,29,30]. If the waste generated from the manufacturing process is not disposed of properly, it will be a major reason for serious health and environmental hazards [31,32,33,34,35]. There has been growing interest in adding together traditional and non-traditional additives for soil stabilization purposes in recent years. The additives include Portland cement, lime, fly ash, bitumen tar, polymer-based products, calcium chloride, and sodium chloride [36,37,38,39,40,41]. The addition of admixture, such as lime and cement, can improve the physical properties of natural clayey soils. In the construction industry, the demand for lime and cement has increased, increasing their cost. Therefore, long-time efforts have been carried out to use waste from the manufacturing process as admixture [42,43,44,45,46,47].

Numerous researchers have conducted studies to determine efficient techniques to reduce the pollution caused by these materials, including by recycling and reusing them in civil engineering applications as a way to protect the environment from waste material pollution [48,49,50,51,52,53]. A practical application of these materials is as a soil stabilizer in road construction. Traditional soil stabilizers, such as cement and lime, are frequently employed to strengthen weak soils’ geotechnical characteristics [54,55,56,57,58]. Numerous research has verified these materials’ efficiency in altering the characteristics of soils [59,60,61]. However, due to their widespread use, these materials are not cost-effective [62,63,64,65,66]. As a result, numerous researchers look for more cost-effective soil stabilizers, such as plastic, tire chips, and rice husks.

Recent and past studies have subjected soils to various treatments; however, a limited number of studies are available for controlling the flow of water within the soil to minimize the effect of structural failures due to soil permeability. Therefore, this study aims to determine compaction characteristics and soil permeability of stabilization with rice husk ash, saw dust, marble dust, and rice husk. In this research, laboratory tests, modified Proctor tests, and permeability tests were conducted to determine the effect of local waste materials on enhancing the geotechnical properties of expansive soil.

## 2. Materials and Methods

### 2.1. Laboratory Tests

The experimental research comprised of adding varying percentages of marble dust, rice husk (RH), rice husk ash (RHA), cow dung ash (CDA), saw dust, and wheat straw to a mixture of oven-dried soil with various percentages (0, 2, 4, 6, 8, and 10%). The process was repeated until the soil and stabilizers were homogeneous in color and texture. The compaction tests were performed on treated and untreated soil samples, in accordance with ASTM 1557 [67].

**Figure 1:**
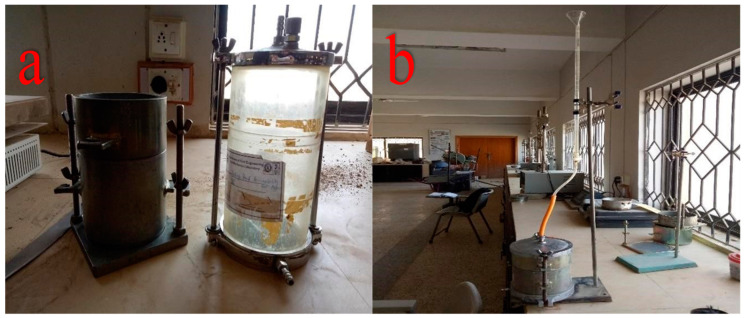
(**a**) Permeability apparatus and (**b**) modified permeability apparatus.

Permeability coefficient (also known as hydraulic conductivity) k is defined as the water discharge rate under laminar flow over a unit cross-sectional area of soil under 20 °C temperature conditions and a unit hydraulic gradient. Flexible wall permeameter equipment was used to perform constant head permeability measurements according to the ASTM D5084 method A (Figure 1) [68].

### 2.2. Expansive Clay

**Figure 2:**
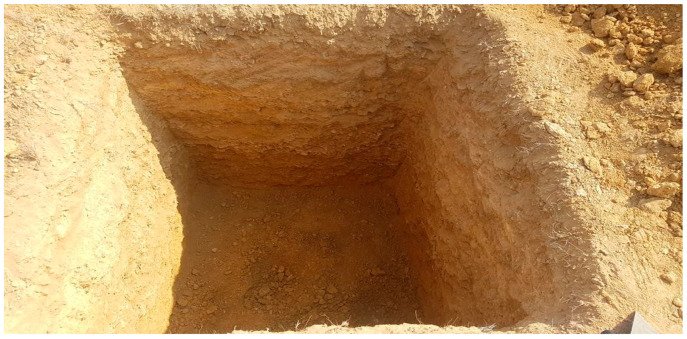
Pit Test.

**Figure 3:**
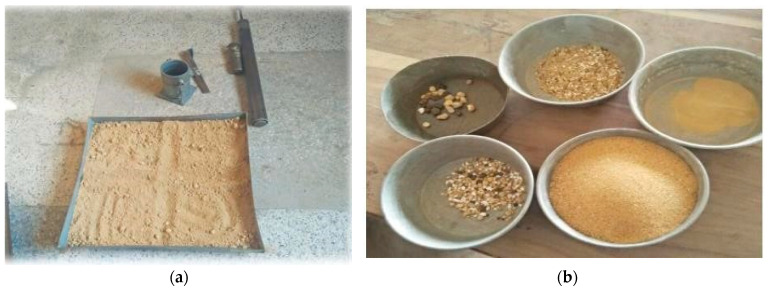
(**a**) Quartering of representative sample (**b**) Sieved samples.

A test pit (Figure 2) excavates the soil to test and collect soil samples from, typically excavated during a site investigation. It is not convenient to take just one sample from the bulk as that sample may not represent the whole bulk. Therefore, a proper procedure is adopted to obtain a representative sample, representing the whole bulk of soil. In our research, we have chosen the quartering method to obtain the required sample, as shown in Figure 3.

### 2.3. Marble Dust

Marble dust is produced during the cutting and polishing of marble stones [69,70,71]. Marble dust was gathered for this study from Hyderabad marble factories. In this study, the waste materials were added to the expansive soil of Jamshoro to observe the effect on the compaction characteristics of soil. The general chemical composition of marble dust is mentioned in Table 1.

### 2.4. Rice Husk and Rice Husk Ash

Rice husk is a common agricultural waste item. Rice husks are burned to remove the cellulose and lignin, leaving just silica ash. Rice production fell to 6900 thousand tons in 2014–2015, down from 7005 thousand tons the previous year. Now, 50,000 tons of solid waste are produced. With 1.7 percent rice husk and 16 percent bagasse (residue from sugar mills), daily crop residue is expected to be 225,000 tons, while animal dung is expected to be 1 million tons. Rice is mostly grown in Pakistan’s Sindh and Punjab regions in the interior. The average annual production of rice husk over the last four years is 1828 tons. Rice husk obtained as a by-product is regarded as a waste and is typically discarded as such, causing waste disposal problems and methane emissions. Rice husk burning is expected to remove organic matter, leaving silica in an amorphous state. Al_2_O_3_, CaO, Fe_2_O_3_, MgO, and K_2_O make up the remainder. Silica content in rice husk varies according to the rice variety, soil and climate conditions, prevailing temperatures, and agricultural practices. Rice husk ash, which has a high proportion of silica, combines optimally with binder ingredients to produce a pozzolanic reaction. This occurs as a result of the formation of extra C–S–H (CaOSiO_2_H_2_O), referred to as calcium silicate gel, in many spaces surrounding hydrated cement particles, so that silica can alter the compressive and flexural strengths [72]. The rice husks were collected from Hyderabad (25.36228, 68.38201).

### 2.5. Cow Dung Ash

Cow dung (CD) is the feces of bull, cow, heifer, and veal, and has generally been used with soil as animal manure [73,74,75]. Cow dung cakes were collected from villages and burned at higher temperatures.

### 2.6. Saw Dust

Saw dust is a by-product produced when wood is cut or pulverized with a saw or other blade in sawmill or lumbering industries. Dry wood is composed primarily of cellulose, lignin, and hemicelluloses, with minor amounts (5–10%) of other components. These components exhibit cementitious characteristics. Trees are required in great numbers for a variety of reasons, and their removal results in a high proportion of saw dust being produced. The fine saw dust was collected from sawmills in the vicinity of the Hyderabad area.

### 2.7. Wheat Straw

Wheat straws are usually accessible after separating the wheat from the straw using thresher machines. After being dried in the sun, the wheat straw fibers were cut into approximate dimensions. Wheat straw is a lightweight material with a high water absorption rate (250%), as seen by its specific gravity and water absorption ratio.

**Figure 4:**
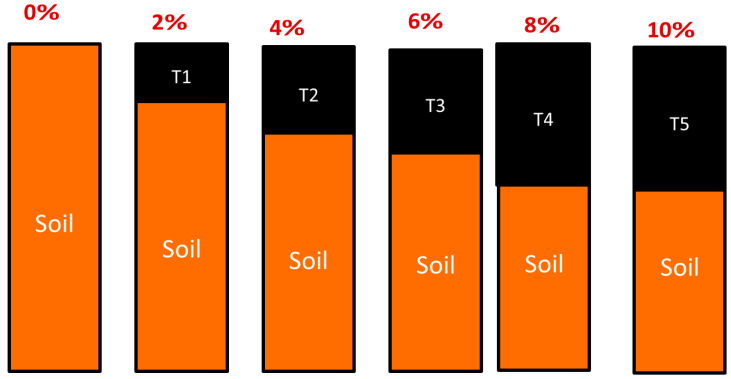
Percentages of waste materials as stabilizer.

All these stabilizers were added to the soil from 0 to 10% (Figure 4) to observe the changes in the liquid limit, modified Proctor, and soil permeability. These percentages were arbitrarily chosen; similar portions of stabilizers were added to the soil in the study of Eliaslankaran et al. [72].

## 3. Results and Discussion

**Figure 5:**
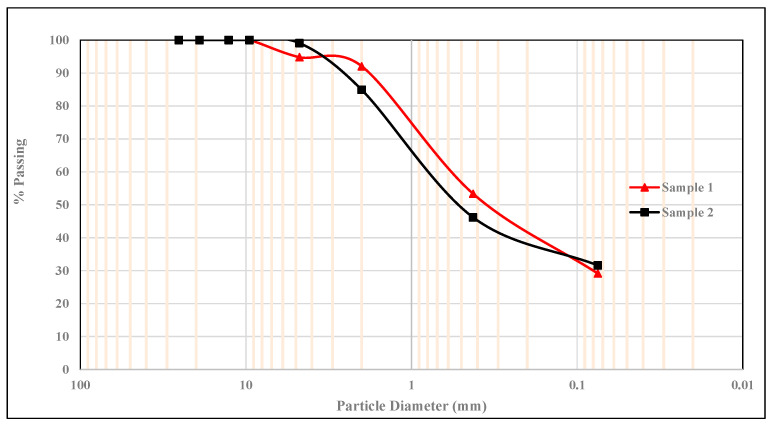
Sieve analysis of soil sample.

The expansive soil is classified as A-7-5, according to the AASHTO soil classification system (AASHTOT 27). Figure 5 shows the particle size distribution curve of soil.

### 3.1. Compaction Characteristics of Stabilized Soil

**Figure 6:**
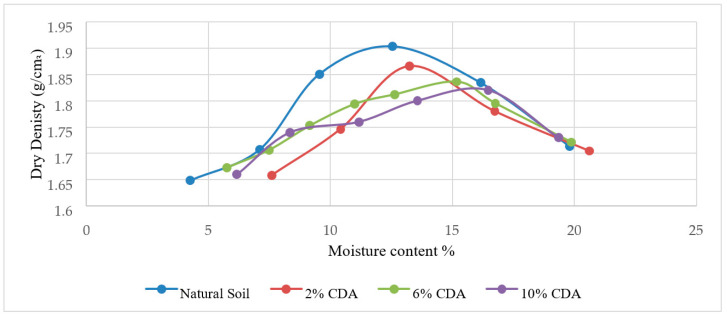
Cow dung ash Proctor results.

**Figure 7:**
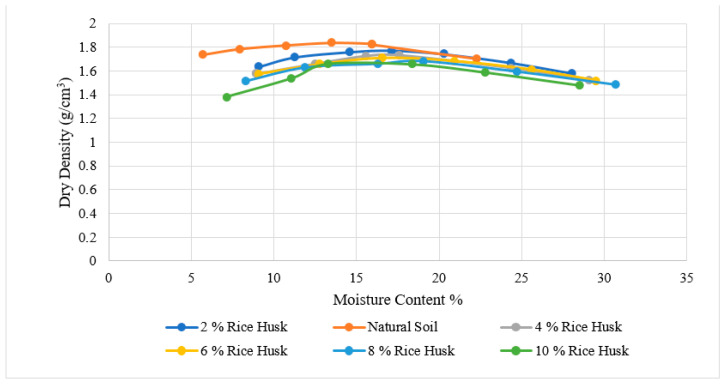
Rice husk Proctor results.

**Figure 8:**
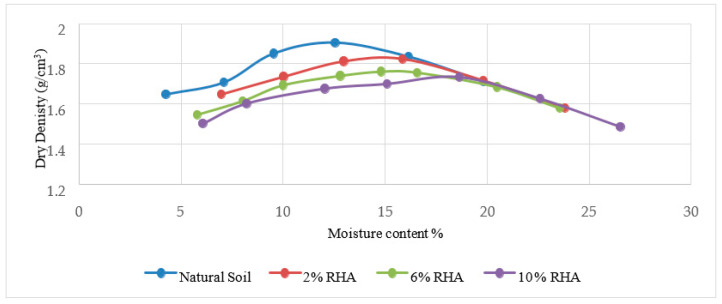
Rice husk ash Proctor results.

**Figure 9:**
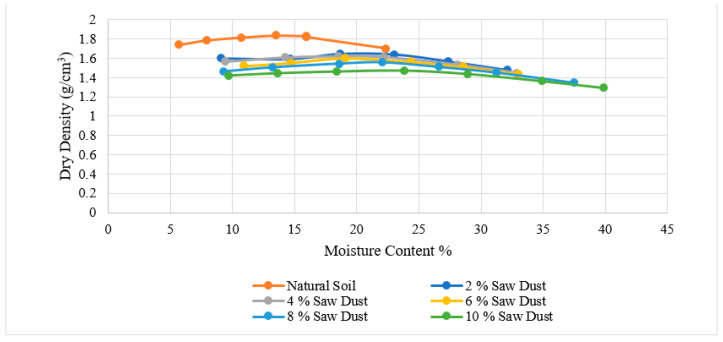
Saw dust Proctor results.

**Figure 10:**
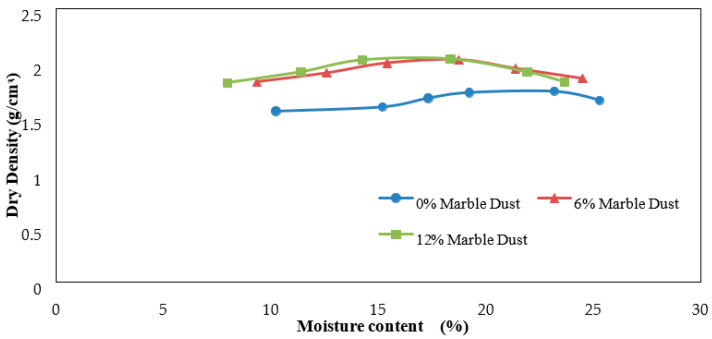
Marble dust Proctor results.

**Figure 11:**
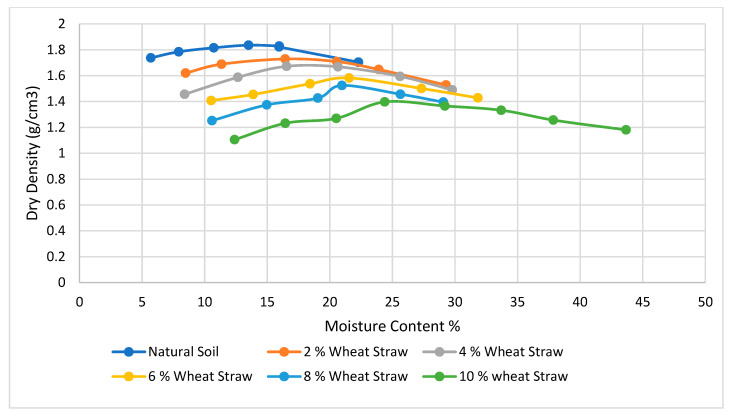
Wheat straw Proctor results.

Figure 6, Figure 7, Figure 8, Figure 9, Figure 10 and Figure 11 shows the variation of maximum dry density and moisture content with waste materials for stabilization. The addition of rice husk, marble dust, saw dust, rice husk ash, and wheat straw to soil results in a decrease in maximum dry density, since all these materials have a low specific gravity. The grains of all the materials replaced denser soil particles (Gs = 2.54), resulting in an overall reduction in the treated soil’s dry density. Moreover, these materials behave as non-plastics, which reduce soil cohesion, thereby reducing soil density. This reduction occurs due to the rapid reactions between the stabilizer and the soil particles (flocculation and agglomeration), resulting in additional voids and a more open structure; hence, decreasing the maximum dry density [76]. Al-Khalili et al. [77] demonstrated that introducing silica fumes increased the optimum moisture content (OMC) from 28.92 to 29.74 percent, and decreased the maximum dry unit weight (MDD) from 14.05 to 13.55 kN/m^3^. The rise in the OMC occurred because the addition of the silica fume increased the soil’s surface area. As the stabilized soil has lower density, it can be used as lightweight fill materials compared to traditional materials, such as gravels and sand.

The MDD decreased from 1.90 to 1.83 with the addition of CDA (cow dung ash), 1.90 to 1.73 with the addition of RHA (rice husk ash), 1.82 to 1.65 with rice husk (RH), 1.82 to 1.47, 1.73 to 1.43 with wheat straw, and increased from 1.73 to 2.04 g/cm^3^ with marble dust. The MDD decreased in all cases except the increase with marble dust. The decrease in dry density leads to an increase in soil permeability; therefore, the swelling of expansive soil decreases [78]. According to [79], increasing the density value increases the number of soil particles contained within a given soil volume, increasing the particle’s specific area. Therefore, the sample’s ability to absorb water and its swelling percentage also increased. As with the addition of all of the stabilizers, the dry density decreased; therefore, the swelling expansive reduced.

### 3.2. Permeability of Stablised Soil

The capacity of soil to enable water to flow under a hydraulic head is referred to as permeability. Permeability is a key characteristic in subsurface flow studies [80]. In terms of soil permeability properties, it is still hard to measure and predict them. The soil permeability is influenced by the porosity, the fabric, the density, and the sediment composition [81]. The apparatus provided porous stone at the end pieces of the specimen, which was contained by a flexible membrane that was sealed at the cap and base. There was constant monitoring, measuring, and recording of the amount of inflow and outflow, as well as of the variations in specimen height. To complete the permeation test, the test was not conducted until the permeability coefficient was stable. The permeability coefficient is calculated using Equation (1):

*k* = *Ah*Δ*t* (1)

where *k* = the permeability coefficient (m/s), ΔQ = the quantity of flow for interval time Δ*t*, the inflow and outflow average (m^3^), L = the specimen’s length (m), Δ*t* = the time interval (=*t*1 − *t*2) over which the flow occurs ΔQ (s), *t*1 = the time at the start of the permeation trial (s), *t*2 = the time at the end of the permeation trial (s), *A* = the specimen’s cross-sectional area (m^2^), *h* = the head loss of water across the specimen (average value) (=(*h*1 + *h*2)/2) (m), and *h*1 = the head loss at *t*1 (m), *h*2 = head loss at *t*2 (m).

Pulverized clay shale was sieved using a no. 4 test sieve. The specimens were produced in different percentages of additives, which amounted to 2%, while the dry weight of soil amounted to 10% by. All specimens were produced in untreated and treated soil at respective MDD and OMC. The samples were prepared according to ASTM D421 and compacted around 0.95γ_dmax_ with an optimum water content. The permeability of stabilized soil was determined after the saturation of the soil sample.

With rising swelling pressure, the hydraulic conductivity of the clay mineral material diminishes; the research indicates a very high link (correlation coefficient) between the two parameters. Because of this finding, it is assumed that the clay sample with a high swelling pressure tends to fill voids; therefore, the hydraulic conductivity value is low. Additionally, it has been proposed that the hydraulic conductivity may be determined only by the swelling pressure without doing the permeability test. The hydraulic conductivity can be determined solely by the swelling pressure without performing the swelling pressure test.

Table 2 shows the variation in permeability of the soils mentioned, with the addition of stabilization materials from 0 to 10%. In all permeability tests of stabilized soil, the improvement was observed. The stabilized soil is considered to be moderately permeable. The stabilized soil has less permeability, so it can be used as a seepage barrier. The experimental study of Patil et al. [82], with the addition of rice husk ash and fly ash in clayey and silty soil, found that permeability improved, and mentioned that this increase is due to the increase in the voids ratio and saturation. Moreover, they added the rice husk ash and fly in higher percentages, compared to the present study. Khudyakova et al. [83] discovered that combining 10% and 20% of incinerator fly ash with soft marine clay increases the permeability by up to seven days. After a period, cementitious and pozzolanic gels form, filling the spaces created by flocculated soil particles. Ghosh and Subbarao [84] observed a similar trend in the stabilization of a low lime fly ash with lime and gypsum. Osinubi [85] and Deb and Pal [86] observed a similar trend in fly ash for direct falling head permeability; Porbaha et al. [87] also observed a similar trend in indirect calculation from consolidation. Show et al. [88] observed an increasing trend in compacted fly and bottom ash mixture with a higher percentage of fly ash. Kim et al. [89] observed a similar trend in a high volume fly ash cement paste composite, composed of various combinations of fly ash, cement, lime, silica fume, and chemical admixtures. Kalkan and Akbulut [90] observed a similar trend in the application of silica fume for natural clay liners.

Wong et al. [91] discovered that peat soil stabilized with a blend of ordinary Portland cement, crushed granulated blast furnace slag, and siliceous sand was capable of decreasing its initial permeability as the curing time increased. They discovered that the coefficient of the permeability reduction in stabilized peat soil is dependent on several characteristics, including fluid viscosity, pore size distribution, grain size distribution, void ratio, and degree of saturation. The structure of clayey soils significantly affects the coefficient of permeability. Additionally, the ionic concentration and thickness of water layers adhered to the clay particles significantly affect the permeability of clays.

## 4. Conclusions

In the present study, the soil was treated with locally available waste materials, such as cow dung ash, marble dust, rice husk, rice husk ash, and saw dust, for compaction characteristics and soil permeability. The permeability of soil was determined by a modified permeameter apparatus. Based on the current experimental study, the following conclusions are drawn:

- The soil is classified as -7-5 according to AASHTO, and CH is based on the USCS classification system. The soil has a higher liquid and plasticity index, and, as the liquid limit increases, so does the swelling potential.
- The maximum dry density decreased, and the optimum moisture increased irrespective of the type of additive, except for an increase in the case of marble dust. This is due to low specific gravity and the non-plastic nature of additives.
- The permeability of soil increased with the addition of saw dust, wheat straw, and rice husk, and decreased with marble dust. The addition of 8% of rice husk ash significantly increased in permeability of the soil observed.
- The decrease in dry density and increase in permeability of the stabilized soil favors the reduction in the swelling potential of expansive soil.
- All these locally available waste materials positively impact the engineering properties of expansive clayey soil. For more favorable results, a higher percentage of these materials should be used. It can be used to build roads, especially low-cost, temporary highways, as well as earth fill.

## Figures and Tables

**Table 1:** Chemical composition of marble dust.

Oxide Compounds	Marble Dust Percent by Weight
SiO_2_	26.53
MgO	18.31
CaO	38.45
Fe_2_O_3_	13.70
Al_2_O_3_	0.39

**Table 2:** Variation in permeability of treated soil.

NAME	Permeability cm/s	Compaction %	Proctor Dry Density g/cc
Natural Soil	2.31 × 10^−^^5^	94.56	1.84
2% Rice Husk	2.36 × 10^−5^	90.40	1.77
4% Rice Husk	2.78 × 10^−5^	89.02	1.73
6% Rice Husk	2.88 × 10^−5^	91.23	1.71
8% Rice Husk	3.11 × 10^−5^	92.26	1.68
10% Rice Husk	3.41 × 10^−5^	90.57	1.66
2% Wheat Straw	2.04 × 10^−5^	93.22	1.73
4% Wheat Straw	2.12 × 10^−5^	95.21	1.67
6% Wheat Straw	2.37 × 10^−5^	92.41	1.58
8% Wheat Straw	2.41 × 10^−5^	90.79	1.52
10% Wheat Straw	2.46 × 10^−5^	89.29	1.40
2% Saw Dust	1.84 × 10^−5^	93.94	1.65
4% Saw Dust	1.89 × 10^−5^	91.02	1.67
6% Saw Dust	1.90 × 10^−5^	93.13	1.60
8% Saw Dust	2.06 × 10^−5^	91.67	1.56
10% Saw Dust	2.52 × 10^−5^	93.88	1.47
2% Rice Husk Ash	4.62 × 10^−5^	92.56	1.81
4% Rice Husk Ash	5.76 × 10^−5^	91.44	1.75
6% Rice Husk Ash	7.115 × 10^−5^	90.33	1.65
8% Rice Husk Ash	3.15 × 10^−4^	93.21	1.63
10% Rice Husk Ash	5.33 × 10^−4^	92.67	1.49

## Data Availability

The data for this study is available on the special request from all corresponding authors.
